# Parasite Viability as a Measure of *In Vivo* Drug Activity in Preclinical and Early Clinical Antimalarial Drug Assessment

**DOI:** 10.1128/aac.00114-22

**Published:** 2022-06-21

**Authors:** Georges F. R. Radohery, Annabelle Walz, Christin Gumpp, Mohammed H. Cherkaoui-Rbati, Nathalie Gobeau, Jeremy Gower, Miles P. Davenport, Matthias Rottmann, James S. McCarthy, Jörg J. Möhrle, Maria Rebelo, Claudia Demarta-Gatsi, David S. Khoury

**Affiliations:** a Kirby Institute, University of New South Wales, Kensington, New South Wales, Australia; b Department of Medical Parasitology and Infection Biology, Swiss Tropical and Public Health Institute, Basel, Switzerland; c University of Basel, Basel, Switzerland; d Medicines for Malaria Venture, Geneva, Switzerland; e QIMR Berghofer Medical Research Institute, Brisbane, Queensland, Australia

**Keywords:** artesunate, malaria, *Plasmodium falciparum*, viability, antimicrobial activity, clinical trials, drug activity, preclinical drug studies

## Abstract

The rate at which parasitemia declines in a host after treatment with an antimalarial drug is a major metric for assessment of antimalarial drug activity in preclinical models and in early clinical trials. However, this metric does not distinguish between viable and nonviable parasites. Thus, enumeration of parasites may result in underestimation of drug activity for some compounds, potentially confounding its use as a metric for assessing antimalarial activity *in vivo*. Here, we report a study of the effect of artesunate on Plasmodium falciparum viability in humans and in mice. We first measured the drug effect in mice by estimating the decrease in parasite viability after treatment using two independent approaches to estimate viability. We demonstrate that, as previously reported in humans, parasite viability declines much faster after artesunate treatment than does the decline in parasitemia (termed parasite clearance). We also observed that artesunate kills parasites faster at higher concentrations, which is not discernible from the traditional parasite clearance curve and that each subsequent dose of artesunate maintains its killing effect. Furthermore, based on measures of parasite viability, we could accurately predict the *in vivo* recrudescence of infection. Finally, using pharmacometrics modeling, we show that the apparent differences in the antimalarial activity of artesunate in mice and humans are partly explained by differences in host removal of dead parasites in the two hosts. However, these differences, along with different pharmacokinetic profiles, do not fully account for the differences in activity. (This study has been registered with the Australian New Zealand Clinical Trials Registry under identifier ACTRN12617001394336.)

## INTRODUCTION

In 2020, there were an estimated 241 million cases of malaria worldwide and 627,000 deaths (1). Moreover, progress in international efforts toward malaria elimination has stalled in recent years, with global elimination targets unlikely to have been met in 2020 (1). Of additional concern, parasite resistance to artemisinin-based combination therapies, the most effective antimalarial regimens, is on the rise (2, 3). Hence, there has been a large international effort both to monitor the efficacy of existing antimalarials (4–6) and to develop new drugs (7–16).

A key step in the drug development pipeline is preclinical assessment of candidate antimalarial compounds for safety and effectiveness in preclinical animal models, before assessment in humans can begin. In both the preclinical and early-stage clinical development of antimalarials, a key metric to assess a drug’s effectiveness is the speed of parasite clearance after treatment, typically reported as the parasite reduction ratio (PRR) or parasite clearance half-life (17). Drugs that induce rapid clearance of parasitemia are deemed potent, fast-acting antimalarials and prioritized for further development (18). However, a recent study has indicated that this metric may underestimate the speed of action of some drugs where there is a dissociation between parasite killing and clearance of killed parasites (19). It was found that artesunate (the recommended drug for treatment of severe malaria) renders parasites nonviable before they are cleared from circulation by the host (19). Measuring the rate of decline of parasitemia after treatment reflects the rate of host removal of dead parasites rather than the rate at which the drug is killing parasites. Therefore, measures of net parasite clearance may not be ideal for assessing antimalarial drug activity *in vivo* (20) and may confound predictions of the overall drug activity. A more detailed investigation of pharmacodynamic (PD) effect by measuring parasite viability represents a potential method for a more accurate characterization of candidate drugs’ activity. It allows a more sensitive estimate of the maximal killing rate and the concentration required to achieve this killing rate.

Despite the potential utility of assessing parasite viability after *in vivo* drug treatment rather than measuring parasitemia, few studies have explored methods of estimating parasite viability *in vivo* after drug treatment or assessed drug activity based on loss of parasite viability *in vivo* (20). An impediment to the adoption of this method is the labor-intensive requirement for parasite culture. A recent study on parasite viability in human volunteer infection studies (VISs) (19) provided a novel method for quantifying viable parasites that relied on culturing blood samples collected from volunteers after treatment. Based on the time for parasites in these cultures to grow above the detection level combined with a mathematical model, it was possible to estimate parasite viability (19) (which here we refer to as the “regrowth” assay). However, an alternative assay that would provide additional validation of this approach was not considered. In this study, we first further validated the recently developed approach for estimating parasite viability after drug treatment in a humanized mouse model using a limiting dilution assay (LDA) as an alternative assay to assess parasite viability. The LDA approach has been used previously to quantify the frequency of viable cells in a diverse range of applications, including after exposing *Plasmodium* parasites to drugs *in vitro* (21). Assessment of parasite viability using the regrowth assay and the LDA in a humanized mouse model of Plasmodium falciparum infection (NOD.Cg-Prkdcscid Il2rg tm1WjI/SzJ) (22) revealed that artesunate acts faster, with a higher maximal killing rate than is evident from the traditional parasite clearance curve, and maintains its efficacy after successive doses *in vivo*. Finally, viability estimates after artesunate treatment from both mice and humans showed that many of the properties of drug activity are consistent between mice and humans, and thus, assessing parasite viability in preclinical models may improve translation potential of these models for predicting drug activity in humans.

## RESULTS

### *In vitro* and *in vivo* validation of parasite viability assay.

**FIG 1:**
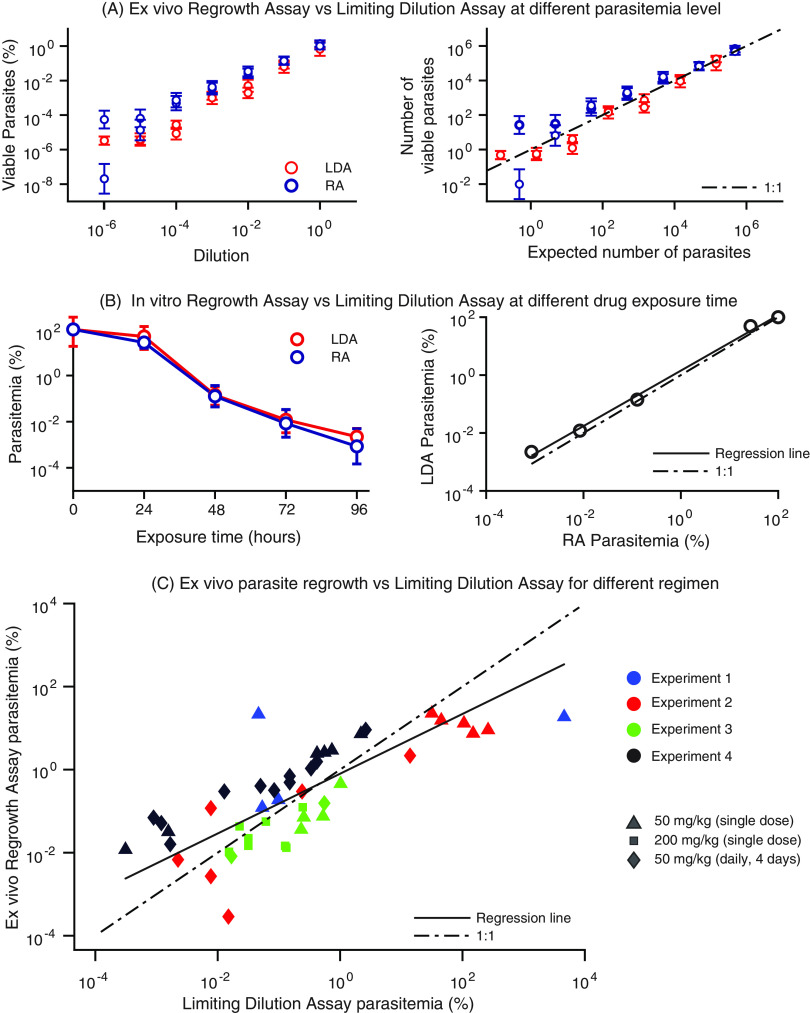
*In vitro* and *in vivo* validation of the regrowth assay (RA) using a limiting dilution assay (LDA). (A) The left panel shows the estimated viable parasites (as a proportion of the viable parasites in the undiluted sample) at different dilutions of the infected red blood cells (RBCs). The right panel shows the estimated absolute number of viable parasites. The dashed diagonal line is the expected number of viable parasites. The red and blue circles are the estimated parasite viability from the LDA and the regrowth assay, respectively. The error bar indicates the 95% CI of these estimates. (B) Comparison of viability estimates from the regrowth assay and the limiting dilution assay after *in vitro* exposure of parasites to artesunate for different exposure times (also known as the parasite reduction ratio [PRR] assay) (21). The left panel shows the parasite viability estimates from the regrowth assay and the LDA normalized to the parasite viabilities estimated after 0-h exposure to the drug. The right panel compares the regrowth assay and the LDA. The dashed line indicates a one-to-one correspondence line. The continuous line represents the fitted correspondence from the parasite viabilities estimated from LDA and regrowth assay. (C) *In vivo* validation of the methods for estimating parasite viability. The figure compares the viability estimates obtained from the LDA and regrowth assay after parasites are exposed to treatment *in vivo* and collected and assessed *ex vivo*. These estimates were obtained using data from four experiments containing a total of 14 mice. The four colors and three symbols represent the four experiments and the three treatments, respectively. The triangles, diamonds, and squares represent the 50-mg*/*kg single-dose, 50-mg*/*kg*/*day 4-dose, and 200-mg*/*kg single-dose regimens, respectively.

To validate the previously published regrowth assay (19) for *in vitro* and *in vivo* assessment of parasite viability, we conducted a series of head-to-head viability assessments of parasites using the regrowth assay and a limiting dilution assay (LDA) approach (19, 21). The methods were compared on three sets of samples: (i) serially diluted untreated *in vitro* samples with known parasitemia, (ii) *in vitro* parasite cultures treated with drug for different durations, and (iii) samples collected from mice after *in vivo* treatment. First, using a serially diluted untreated sample of parasitized red blood cells (RBCs), we performed pairwise limiting dilution and regrowth assays to estimate the viable parasite concentration of the serially diluted samples. Results obtained with the two methods agreed closely (Fig. 1A and Fig. S1), with some divergence in the regrowth assay toward higher viability estimates at low concentrations (Fig. 1A). It was noticeable that both assays allow quantification of parasites at near 1 parasite per sample cultured, suggesting these are highly sensitive assays. Second, we performed an *in vitro* drug exposure assay (also known as parasite reduction ratio [PRR] assay [21]), in which parasites were exposed to artesunate for different durations (21). The viability of parasites after different durations of drug exposure was then assessed using the regrowth assay and LDA. Again, both approaches yielded consistent estimates of parasite viability (Fig. 1B). Finally, we compared the regrowth and LDA methods for assessing viability of parasites after artesunate treatment *in vivo* in P. falciparum-infected NSG mice. We estimated parasite viability after treatment using both a regrowth assay (Fig. S2) and LDA across four repeat experiments with three different dosing regimens (total of 63 time points sampled with both assays across 14 mice). The results showed that parasite viabilities estimated by the two approaches agree for most samples (Fig. 1C). However, we found a slight bias toward higher parasite viability estimates with the regrowth assay at low parasite numbers. Together, these findings suggest that parasite viability estimates are broadly consistent when evaluated in the regrowth assay and LDA after drug treatment *in vitro* and *in vivo*. Consequently, in the following analysis we decided to consider only the regrowth assay results for consistency with the recently reported study in human volunteers (19).

### Viable parasite numbers decline faster than parasitemia after treatment in mice consistent with observations in humans.

**FIG 2:**
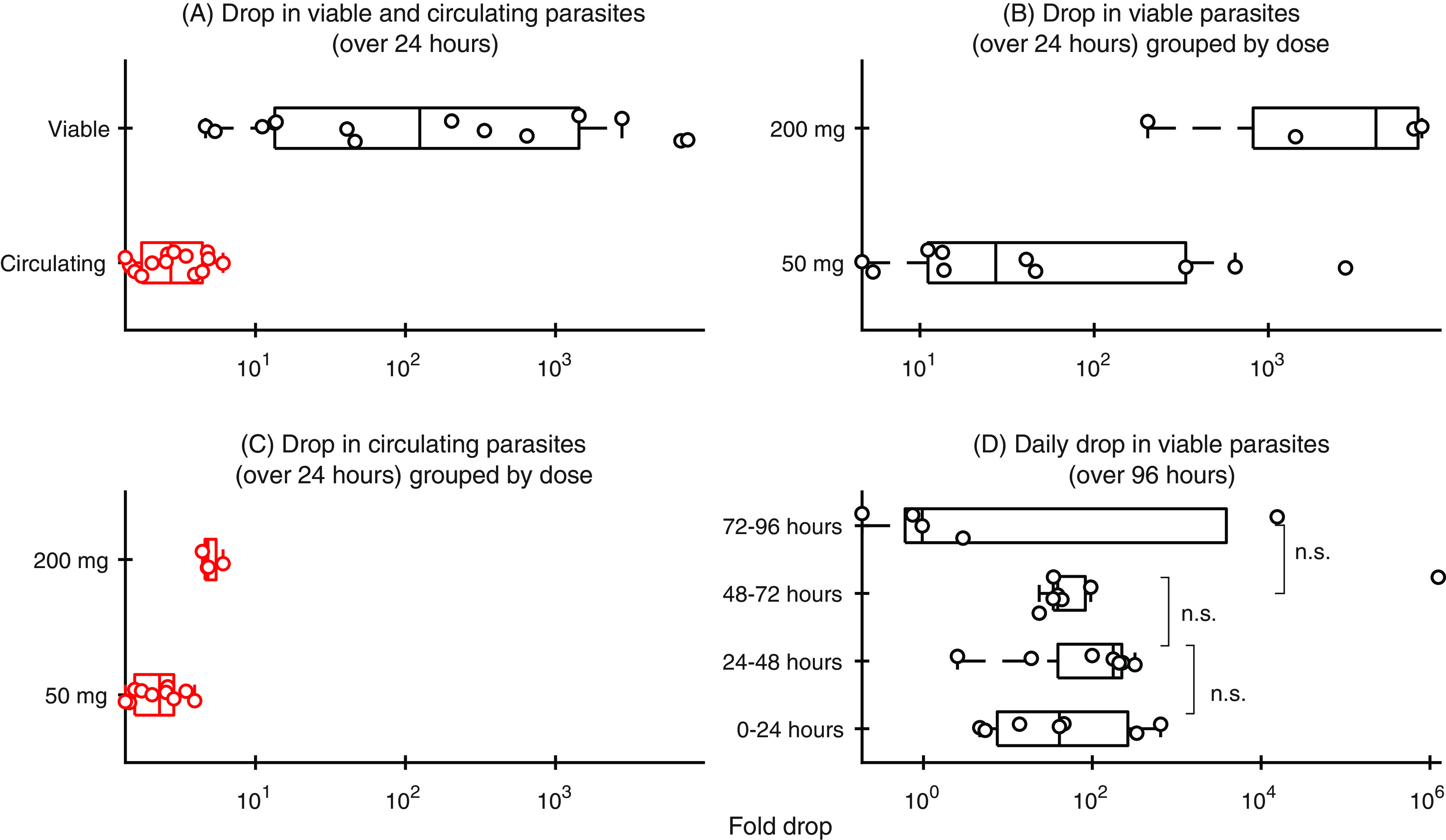
Panel A shows the comparison of the drop in the number of circulating parasites (red) and the number of viable parasites (black) at 24 h posttreatment for all treatment groups. Panels B and C show the comparison of the drop in the number of circulating parasites and the number of viable parasites at 24 h posttreatment grouped by treatment. (D) Daily fold drop in viable parasite numbers following each of the daily doses of 50 mg/kg for 4 days.

Previously, it was shown in humans that viable parasite concentrations decline much faster than total parasitemia after treatment with artesunate in experimentally infected volunteers (19). Here, we tested whether the same was true in P. falciparum-infected NSG mice. We found that the number of viable parasites drops 467-fold (95% confidence interval [CI], 91, 954), more than the number of circulating parasites in mice, at 24 h posttreatment (Fig. 2). This suggests that, like in human infection volunteers (19), the maximum killing rate of artesunate is faster than is indicated by the parasite clearance curve.

### Maximal killing rate achieved at very high doses of artesunate.

Parasite clearance rates in some murine models typically begin to saturate at artesunate doses of 50 mg/kg of body weight, with only modest improvement in clearance achieved with higher doses (23). Here, we investigated if increased drug activity is evident at 200 mg/kg by assessing drug activity based on parasite viability rather than total parasite clearance. We found that increasing the dose from 50 to 200 mg/kg causes a 2.2-fold (95% CI, 1.6, 2.8) increase in the decline in total parasitemia over the first 24 h, but there is a 23.9-fold (95% CI, 1.9,130.7) increase in the decline in viability over the same period (Fig. 2B). This finding shows that, although only a marginal improvement to the drop in parasitemia is observed by increasing the dose from 50 and 200 mg/kg, a much larger effect is observed in the reduction of parasite viability.

### Effect of subsequent artesunate doses on parasite viability and recrudescence.

**FIG 3:**
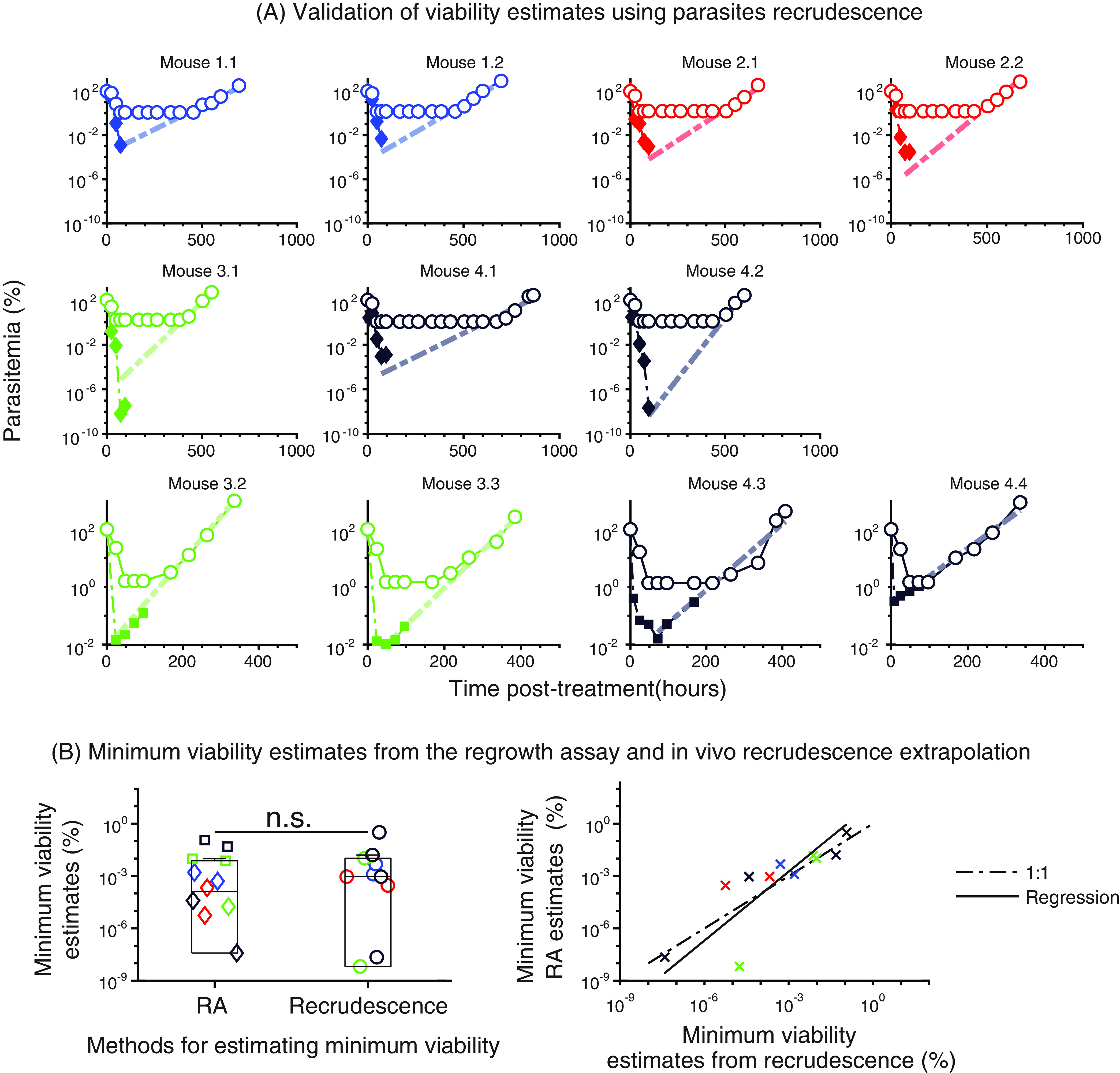
Validation of parasite viability estimate from the regrowth assay (RA). Panel A shows the parasite viability estimates from the RA. Mice are designated as follows: mouse 1.1 is the first mouse treated from experiment 1, mouse 1.2 is the second mouse treated from experiment 1, mouse 2.3 is the third mouse treated from experiment 2, etc. Treatment 1, (top two rows, diamonds) 50 mg/kg/day for 4 days; treatment 2, (bottom row, squares), single 200-mg/kg dose. Note that mice treated with a single 50-mg/kg dose of artesunate were not included in this analysis because cure (parasitemia below the limit of detection) was not consistently achieved, and thus estimating recrudescence kinetics was not possible. The circles represent the measured circulating parasites. Each color represents an experiment. (B) Comparison of the minimum parasite viability estimated from the regrowth assay and the number of viable parasites that could lead to the recrudescence we saw in the mice.

Given the high sensitivity of the viability assay to detect viable parasites at very low densities (Fig. 1A), we used the regrowth assay to determine whether subsequent doses of artesunate had a greater or smaller killing effect than the first dose. If, for example, there is a less-susceptible subpopulation of parasites that can survive sequential artesunate doses, this should manifest as a reduced killing effect. To explore this question, we treated mice with four sequential daily doses of 50 mg/kg, collected blood samples 24 h after each dose, and assessed viability using the regrowth assay. We found that the fold change in parasite viability following each of the four successive doses were similar, although considerable variability between notionally identical mice with identical treatments was also present (Fig. 2C). There was a slight trend toward a lower killing effect after the fourth dose, but this was not significant (Fig. 2C). Moreover, we found that the parasite viability estimates were consistent with the emergence of recrudescent parasites (Fig. 3). Therefore, we did not find strong evidence of a diminishing effect of artesunate with each subsequent dose, and rates of overall killing assessed by parasite viability and recrudescence *in vivo* were consistent.

Interestingly, despite the faster drop in viability in the first 24 h after treatment with 200 mg/kg of artesunate compared with 50 mg/kg (as shown in Fig. 2B), four separate doses of 50 mg/kg resulted in a 77-fold (95% CI, 6, 430) greater overall drop in parasite viability (by comparing the minimum viability after four daily doses of 50 mg/kg with minimum viability after a single 200-mg/kg dose). This suggests that a trade-off exists between speed of activity (achieved at higher doses) and total overall killing activity (achieved with regular lower doses).

### Consistent kinetics of parasite viability in mice and humans but evidence of some pharmacodynamic differences.

Under the existing measure of drug activity (i.e., parasite clearance rates), the drug activities in P. falciparum-infected NSG mice and humans have been observed to differ—including after treatment with artesunate (23). Here, we considered whether the human volunteer infection study (VIS) (19, 24) and humanized mouse models are more congruous when parasite viability is considered instead of the traditional parasite clearance estimates. To do this we combined previously published data on parasite viability after artesunate treatment from a VIS (19) with the data obtained in P. falciparum-infected NSG mice treated with the same compound. We applied a pharmacometric model to relate killing (loss of parasite viability) to *in vivo* drug concentrations (see Materials and Methods and the supplemental material). We assessed whether the differences in total parasite killing observed in mice and humans treated with artesunate could be explained solely based on the differences in pharmacokinetics (PK) of artesunate and its active metabolite, dihydroartemisinin (DHA), in the different hosts.

We used a one-compartment open model to characterize the pharmacokinetic profile of DHA in the mice (*n* = 4) and the human volunteers (*n* = 5 [using data from reference 24]), as described in references 25 and 26. Table 1 summarizes the pharmacokinetic (PK) model parameters that we obtained from fitting this model to data on the DHA concentrations measured in four mice (see Fig. S3 and S4 in the supplemental material). Given limited existing DHA PK parameter estimates in the preclinical mouse model, we also compared these PK parameter estimates to other published studies on mice (Table 1). We noticed that DHA has similar half-lives in both mice and humans (Table 1), despite much higher doses (per kilogram of body weight) of artesunate being required to achieve high DHA concentrations in mice than in humans.

**TABLE 1:** DHA PK parameters estimated from mice treated with a dose of 50 mg/kg/day for 4 days and mice treated with a single dose of 200 mg/kg^*a*^

Parameter	Population estimate (95% CI) for:		Mouse parameter estimate from literature (reference)
	Mice	Humans	
*V_d_*/*F* (L)	0.55 (0.39, 0.71)	165.9 (132.7, 199.2)	0.91^*b*^ (50), 0.85 (51)
CL/_F_ (L/h)	0.94 (0.73, 1.15)	254.5 (144.5, 364.5)	1.53^*b*^ (50), 1.98 (51)
*t*_1/_*_2_* (h)	0.40^*c*^	0.45^*c*^	0.41 (50)

a *V_d_*/*F*, apparent volume of distribution; CL/*F*, apparent clearance; *t*_1/2_, half-clearance time. The DHA PK parameter estimates were obtained by fitting a one-compartmental model to the human data from reference 24. We note that PK parameters from this study have previously been reported in the original study using a noncompartmental analysis (24). Thus, we performed a comparison of the parameters obtained here from a one-compartmental model from the human volunteer infection study with the parameters reported using the noncompartmental parameters in the original study (24) (see Table S1 in the supplemental material). Differences in the PK parameters observed in the original clinical trial and the analysis performed here (Table S1) likely arise from the use of a compartmental PK model in this study compared to a noncompartmental model in the original report and because the analysis in this article focuses on only a subset of individuals from the original trial (*n* = 5 of a total of 22) in whom parasite viability was assessed.

b Estimates assume an average mouse weight of 25 g.

c Half-life computed from the clearance (CL) and the volume of distribution (*V_d_*): $t_{1/2}=\ln2\frac{V_{d}}{\text{CL}}$.

**FIG 4:**
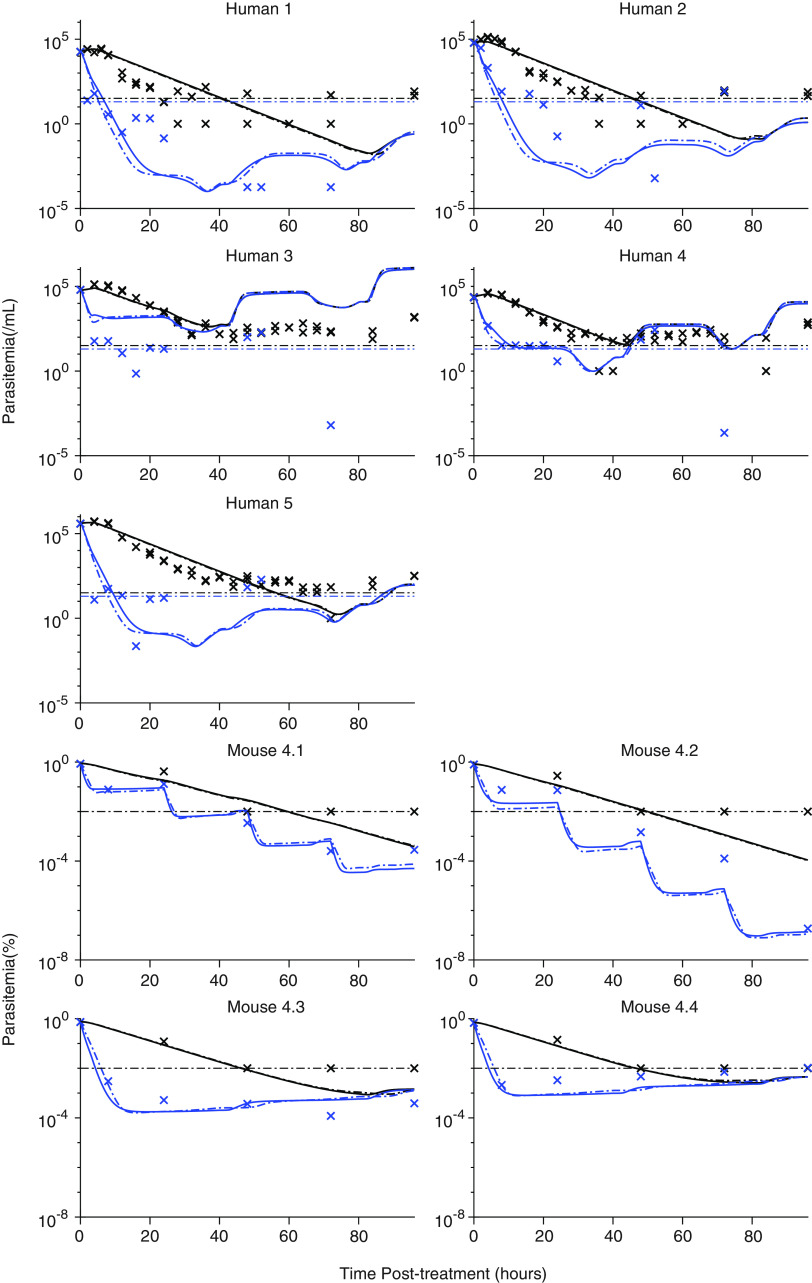
Model of drug effects in humans and mice. The total circulating parasite concentration (black) and viable parasite concentration (blue) are shown for each human and mouse (designated as described in the legend to Fig. 3) infected with P. falciparum 3D7 parasites and treated with artesunate (human data from reference 19). The crosses represent the measured data. Viable and total parasite concentrations are reported as parasites per milliliter in humans and as a percentage of initial parasitemia in mice. Measurements of parasite concentration in humans were performed in triplicate. The lines are the fitted model of parasite killing and clearance. The dotted lines are fitted from a model where the maximum killing rates are the same in mice and humans but the EC_50_s are different, while the continuous line represent a model where the EC_50_ is the same, but the maximum killing rates are different between mice and humans. The dotted horizontal lines indicate limits of detection of 20 parasites/mL and 32 parasites/mL for viable and circulating parasites, respectively, and 0.01% parasitemia for circulating parasites in mice.

To model the drug action in both mice and humans, we used a pharmacodynamic (PD) model in which viable parasites are growing at rate *g* (per hour) and killed at rate κ (per hour). The dead parasites are then removed at a separate rate, δ (per hour). The killing rate κ is a sigmoidal function that depends on the DHA concentration in the hosts, a maximum killing rate (*E*_max_), and EC_50_, the DHA concentration needed to reach half of that maximum killing rate. The fitted pharmacokinetic model (Fig. S3) and information on parasite life stages at the time of treatment (see section C in the supplemental material) were combined with this pharmacodynamic model, and the combined PK/PD model was fitted to the human and mouse data on the parasite viability and total parasite concentrations after treatment with artesunate (Fig. 4). Fitting this model provided estimates of the 50% effective concentration (EC_50_) of DHA, the maximal killing rate (*E*_max_), and Hill coefficient (γ), in mice and humans, and we explored whether any of these killing parameters were different between mice and humans. We performed forward model selection to determine which of the PD parameters *E*_max_, EC_50_, and γ were significantly different between humans and mice (Table S2 and Fig. S7). Table 2 summarizes the parameter estimates from the two best PD models. We found that the model with same EC_50_ values in mice and humans (0.0074 μg/L) but different *E*_max_ values (1.45 h^−1^ and 0.74 h^−1^ in humans and mice with corresponding 0.48-h and 0.92-h killing half-lives, respectively) or the same *E*_max_ (1.34 h^−1^) but different EC_50_s (0.0025 μg/L and 1.32 μg/L n humans and mice, respectively) provided comparable fits of the data (Fig. 4) and that these are the best fits to the data. We also found that these estimates do not influence the clearance half-life of nonviable parasites, which is equal to 3.76 h (95% CI, 3.26, 4.34) and 7.08 h (95% CI, 6.19, 8.11) in humans and mice, respectively. The latter observation is consistent with observations of slower parasite clearance after drug treatment in the NSG mouse model compared with human infection (27). Together, these findings suggest that although parasite viability provides a number of consistent observations between the dynamics of artesunate activity in mice and humans, differences in these infection models remain, which could not be completely reconciled by assessing parasite viability.

**TABLE 2:** Two best-fitting pharmacodynamic models^*a*^

Parameter	Estimate	95% CI	AIC
*E*_max_ and γ common to humans and mice			743.70
*E*_max_ (h^−1^)	1.34	(1.04, 1.74)	
δ_H_ (h^−1^)	0.18	(0.16, 0.21)	
δ_M_ (h^−1^)	0.097	(0.085, 0.11)	
EC_50H_ (μg/L)	0.0025	(1.5 × 10^−4^, 0.04)	
EC_50M_ (μg/L)	1.32	(0.22, 7.87)	
γ	0.41	(0.23, 0.73)	

EC_50_ and γ common to humans and mice			741.96
* E*_maxH_ (h^−1^)	1.45	(1.21, 1.73)	
* E*_maxM_ (h^−1^)	0.74	(0.63, 0.87)	
δ_H_ (h^−1^)	0.18	(0.15, 0.22)	
δ_M_ (h^−1^)	0.097	(0.086, 0.11)	
EC_50_ (μg/L)	0.0074	(0.0012, 0.044)	
γ	0.70	(0.26, 1.86)	

a The two best fitting models are shown. The first has a single *E*_max_ (maximum killing rate) and γ (Hill coefficient) between mice and humans, but different EC_50_s (drug concentration to reach the half-maximum killing rate). The second is a model with a single EC_50_ and γ, but different *E*_max_s between mice (*E*_maxM_) and humans (*E*_maxH_). The parameter δ (dead parasite clearance rate) is different between mice (δ_M_) and humans (δ_H_) in both models. These models provided better fits than other models tested, based on nested model comparison and AIC, but have comparable AIC to each other (AIC difference of <10 [see Table S3 in the supplemental material]).

## DISCUSSION

Parasite clearance, defined as the decline in total circulating parasite numbers after treatment, is a key metric for assessment of antimalarial drug activity in preclinical and early clinical trials (20). Such data are used to inform decisions on which agents are fast and slow acting and to inform modeling and dosing schedules in subsequent clinical trials (18). Although it is relatively easy to measure total circulating parasite levels (or parasitemia), this metric describes the decline of the total circulating parasite level and does not distinguish the loss of viable and nonviable parasites (19). This means that it is possible to underestimate the activity of drugs that kill parasites faster than the rate at which the host removes nonviable parasites (19). Measuring parasite viability in a host after drug treatment represents a more accurate means of estimating drug activity *in vivo*. However, despite the potential utility of measures of parasite viability for understanding the *in vivo* activity of novel antimalarial drugs, consideration needs to be given to how best to assess viability. The approach we reported for determining parasite viability in a volunteer infection study involved collecting parasites from individuals after treatment and culturing *ex vivo* until parasite regrowth was detected (“regrowth” assay). This approach relies on modeling parasite regrowth in culture and assumes exponential growth in cultures (19). An alternate scenario is that parasites exhibit dormancy, or a slower growth (even if viable), after removal from drug exposure *in vivo*. This is particularly relevant to artesunate, where dormancy has been reported after treatment (28). To address these alternate scenarios, we employed an alternative approach for estimating viability that was independent of assumptions of exponential growth, namely, a limiting dilution assay, which has been used previously to assess *in vitro* drug activity of different antimalarial drugs (21). These assays were in close agreement and provide very sensitive methods for estimating parasite viability, seemingly able to detect parasitemia at near 1 parasite per blood sample (Fig. 1A). The LDA is a more standard approach for estimating the frequency of viable cells and less dependent on assumptions around whether parasites grow exponentially. It also appears that the regrowth assay has a slight tendency to overestimate viable parasite concentrations at low viable parasite frequencies (Fig. 1). However, the LDA has the additional challenge of requiring many more (>10 times) cultures to be established and maintained per sample analyzed. This is an important consideration: since larger numbers of cultures are required in the LDA, ideally each culture will be performed in smaller volumes than the regrowth assay, so as to, for example, fit on a 96-well plate. However, we note that in the human volunteer infection study, where the regrowth assay was first used, large-volume cultures have been used (in 6-well plates) because *in vivo* parasite concentrations in these participants are very low, and thus smaller-volume cultures are likely to be insufficient for detecting viable parasites. Thus, given the general agreement of the two approaches, the choice of which of these assays is most appropriate in a given study may depend on factors such as the amount of blood required, the expected density of viable parasites, and the number of cultures that can be feasibly maintained.

Measurements of parasite viability provide a more accurate means of estimating a drug’s killing effect than total circulating parasite numbers and provide new insights into the *in vivo* activity of antimalarial drugs. In particular, previous studies with artesunate treatment in mice have found that the decline in parasitemia after treatment is not consistent with the *in vivo* recrudescence of the infection (29). This phenomenon was hypothesized to be explained by the presence of dormant parasites or an otherwise less-susceptible subpopulation of parasites, which may persist at low levels and recrudesce once drug pressure is removed (29). However, by measuring parasite viability in this study, we observed that the *in vivo* recrudescence of infection is consistent with the concentrations of viable parasites achieved *in vivo* (Fig. 3), and this was also true in human infection (19). Further, we do not observe a strong decrease in the killing effect of sequential doses of artesunate—which would be expected if a subpopulation of less-susceptible parasites were surviving each dose. Hence, the apparent discrepancy between the decline in parasitemia and the recrudescence of infection previously observed in this infection model can be accounted for by recognizing that the decline in parasitemia is not an accurate measure of drug killing activity (20).

In the P. falciparum-infected NSG mouse model, we have been able to explore different dosing regimens. The higher single dose of 200 mg/kg achieved a much greater reduction in viable parasite numbers than the lower dose of 50 mg/kg in the first day after treatment (Fig. 2B). Interestingly, this difference was not reflected in the parasitemia decline, which was only marginally higher with the higher dose. This is likely because removal of nonviable parasites was a rate-limiting step in the time for parasitemia to decline, and so parasitemia was not greatly affected even with significant increases in killing. This highlights a potential confounder of relying on the parasitemia as a measure of drug activity, since it is, at least in the case of artesunate, limited by host removal of nonviable parasites rather than the speed of drug action: the maximal killing rate and the dose at which killing saturates may be underestimated. In the case of artesunate, it is clear from human trials studying cure rates that increasing the dose does not necessarily deliver a better overall killing effect, since all parasites are eventually killed and people are cured (30). However, in this study, we have observed that higher doses may cause faster killing of parasites (even if its undetectable by a faster decline in parasitemia) (31). Caution must be used in extrapolating these observations to the clinical setting, since the results in murine infection presented here have not been confirmed in a clinical setting and would require the measurement of parasite viability in infected individuals after treatment with different doses of artesunate. However, we propose that when seeking to determine the maximal killing rate and the dose at which this is achieved in human or murine infection, measuring parasite viability rather than the decline of parasitemia is likely to lead to more reliable predictions.

It was also observed in this murine study that 4 daily doses of 50 mg/kg produced more overall parasite killing and later recrudescence of infection than a single 200-mg/kg dose. This is consistent with clinical observations that have established that artesunate monotherapy requires 7 daily doses to achieve consistent cure in malaria infection (32) and is perhaps unsurprising given the very short half-lives of artesunate and DHA *in vivo*. That is, with a very short half-life for DHA, a large single dose does not greatly prolong the overall amount of time the killing is above the 50% inhibitory concentration (IC_50_) compared to giving the drug more frequently at lower doses (33).

A limitation of this study was the limited data on the pharmacokinetics of DHA in mice, with data from 4 mice included in this aspect of the analysis. Further, the authors identified very limited literature on the pharmacokinetics of DHA in mice (Table 1). The small number of mice for which DHA data was available limited the ability of this study to dissect, for example, any dose-dependent effects on the pharmacokinetics of DHA (with no significant dose effect found [*P* = 0.32 by likelihood ratio test], although the study is not powered to detect this difference). A better understanding of the pharmacokinetics of DHA in mice may shed some additional light on the much larger doses of artesunate required to achieve similar *in vivo* concentrations of DHA in mice and humans.

The development of a P. falciparum-infected humanized mouse model in which mice are engrafted with human erythrocytes (34) has provided a preclinical *in vivo* tool for studying antimalarial drug activity against human parasites (35–38). Although this tool has been widely applied, its ability to predict outcomes in humans has been questioned (39). In particular, the parasite clearance curves in mice and humans are known to differ considerably after treatment with an antimalarial drug, with slower clearance, including with artesunate, usually evident in mouse models (20, 23, 30). It is often assumed that this is because the drug kills less quickly in mice than in humans. However, here we have observed that the reason for slower declines in parasitemia in this humanized mouse model is, at least in part, explained by a slower clearance of nonviable parasites rather than necessarily slower killing of the parasites by artesunate (Table 2). This is possibly attributable to altered splenic filtering function in the NSG mouse. Measuring parasite viability rather than relying on declines in parasitemia will allow us to better appreciate where the NSG mouse model and human results truly diverge. In this study, by assessing parasite viability in mice and comparing it to similar assessments in humans, we have found that the patterns of drug activity are qualitatively similar. However, importantly, we note that there was not complete consistency in the pharmacodynamic parameters observed between the NSG mouse model and human infection. We found that there are likely to be differences in either the EC_50_ or *E*_max_ between mice and humans, but probably not both (see Table S3 in the supplemental material). Despite this difference, it is possible other pharmacometric differences in the two species, which are not measured here, may at least in part explain the apparent differences in the pharmacodynamics of artesunate (and its active metabolite dihydroartemisinin) in mice and humans. For example, a higher apparent EC_50_ in mice may be the result of different drug-host interactions, such as higher protein binding of the compound in mice than in humans. We hypothesize that the apparent differences in the pharmacodynamic parameters reported here for mice and humans may be accounted for by a better understanding of the differences in drug absorption, elimination, bioavailability, protein binding, and host removal of nonviable parasites by the different hosts—rather than a fundamental difference in the drug activity against the notionally equivalent parasite species used in both the human volunteer infection studies and the NSG mouse model (i.e., P. falciparum 3D7).

An alternate method for assessing parasite viability or an improved *ex vivo* growth assay for assessing parasite viability would be desirable as both assays used in this study are labor intensive and only feasible in well-resourced laboratories, making their application less practical for general use in hospital settings where malaria is endemic. However, these methods are sensitive, reliable, and potentially useful tools in preclinical and early clinical stages of drug development. Using these methods more frequently in the appropriate settings is likely to lead to a better understanding of the *in vivo* drug activity of emerging compounds and may contribute to the better translation of preclinical results and improve the design of phase I clinical trials.

## MATERIALS AND METHODS

### Ethics statements.

Studies with animals were performed at Swiss TPH (STPH) in Basel. The studies performed at STPH were approved by the veterinary authorities of the Canton Basel-Stadt (permit no. 2303) based on Swiss cantonal (Verordnung Veterinäramt Basel-Stadt) and national (the Swiss animal protection law, Tierschutzgesetz) regulations. All experiments were carried out in accordance with European Directive 2010/63/EU.

The human biological samples were obtained as part of an exploratory study of a malaria volunteer infection study randomized clinical trial conducted at Q-Pharm Pty., Ltd., Brisbane, Australia, and approved by both the QIMR Berghofer and Australian Red Cross Blood Service Human Research Ethics Committees. The study was registered with the Australian New Zealand Clinical Trials Registry under identifier ACTRN12617001394336.

### Induced blood-stage infection and artesunate treatment in NSG mice.

Compound efficacy was assessed in the murine P. falciparum NSG model, as described by Jimenez-Diaz et al. (38). Briefly, artesunate regimens (4 daily doses of 50 mg/kg, 1 dose of 50 mg/kg, and 1 dose of 200 mg/kg) were formulated in 70% Tween 80 (density = 1.08 g/mL) and 30% ethanol (density = 0.81 g/mL) and administered to a cohort of age-matched female immunodeficient NSG mice (The Jackson Laboratory, Bar Harbor, ME). These mice had been engrafted with human erythrocytes 14 days earlier (generously provided by the Blood Bank in Zürich, Switzerland). Prior to compound treatment, mice were intravenously infected with 2 × 10^5^ P. falciparum Pf3D7^0087/N9^-infected erythrocytes (day 0). On day 3 after infection, mice were randomly allocated to treatments that were administered orally. Parasitemia was measured by microscopy. Chimerism was monitored by flow cytometry using anti-murine erythrocyte TER119 monoclonal antibody (Pharmingen, San Diego, CA) and SYTO-16 and then analyzed by flow cytometry in serial blood samples (2 μL) collected every day until the completion of the experiment. Parasite recrudescence was detected by microscopy.

The concentration of dihydroartemisinin (DHA) in mice (*n* = 2) treated with the 4-daily-dose 50-mg/kg regimen was measured at 0.75, 1.5, 3, 8, 26, 50, and 74 h, and the concentration of DHA in mice (*n* = 2) treated with the 200-mg/kg single-dose regimen was measured at 0.75, 1.5, 3, and 8 h.

### Human volunteer infection study.

The human data used in this study were obtained from a randomized clinical trial (ACTRN12617001394336) (24) and the associated exploratory viability study (19). Briefly, 5 volunteers were infected by intravenous injection of blood-stage 3D7 P. falciparum parasites. The volunteers were administered a single dose of artesunate monotherapy (2 mg/kg) 8 days postinfection. Parasitemia in the volunteers was measured by quantitative PCR (qPCR) twice per day before treatment and at least every 4 h in the first day following artesunate administration. The concentrations of artesunate and dihydroartemisinin (DHA) in blood were determined at time points 0.25, 0.5, 1, 1.5, 2, 2.5, 3, 4, 6, 8, 10, and 12 h (24).

### *In vitro* validation of LDA and regrowth assay. (i) Validation under drug-free conditions.

The ability of the LDA and regrowth assay to quantify parasite viability *in vitro* was first validated by comparing their viability estimates to a serial dilution of parasites with known parasitemia. In brief, a Plasmodium falciparum Pf3D7^0087/N9^ culture was adjusted to 0.5% parasitemia and 5% hematocrit using microscopy. The culture was centrifuged (5 min at 600 × *g*), and 100 μL of the red blood cell (RBC) pellet was six times 10-fold serially diluted in uninfected RBCs, yielding a total of seven parasite dilutions. Each dilution was split into two 45-μL aliquots, one being used for the LDA and one for the regrowth assay.

For the *in vitro* regrowth assay (IRA), 10-μL duplicates of each parasite concentration were further diluted 1:10 in RBCs and transferred to a well of a 24-well plate filled with 1,900 μL RPMI medium supplemented with hypoxanthine (50 mg/L), HEPES (5.94 g/L), Albumax (5 g/L), sodium bicarbonate (2.1 g/L), and neomycin (100 mg/L). Cultures were maintained at 37°C with 93% N_2_, 4% CO_2_, and 3% O_2_ in humidified modular chambers, and parasite growth was monitored by flow cytometry until parasitemia reached 2%. Medium was replaced on Mondays, Wednesdays, and Fridays, and fresh RBCs were provided once a week.

For the LDA, 10 μL of each parasite concentration was further diluted in RBCs and medium to reach 500 μL of suspension at 5% hematocrit. In a 96-well plate, aliquots of 200 μL were 15 times 4-fold serially diluted in duplicate. Plates were maintained at 37°C and 93% N_2_, 4% CO_2_, and 3% O_2_ in humidified modular chambers for 21 days. Medium was replaced on Mondays, Wednesdays, and Fridays, and fresh RBCs were provided once a week. Parasite growth was measured with [^3^H]hypoxanthine added after 18 days of cultivation.

### (ii) Validation under drug exposure.

A Plasmodium falciparum Pf3D7^0087/N9^ culture was adjusted to 0.6% parasitemia and 5% hematocrit using microscopy. The culture was exposed to 90 nM artesunate for up to 96 h; drug and medium were replenished once a day. A sample of 1 mL was collected every 24 h, and drug was removed by washing three times (centrifugation for 5 min at 600 × *g*).

For the IRA, the 10-μL pellet of the washed sample was processed as described for drug-free conditions in duplicate.

For the LDA, the 20-μL pellet of the washed sample was diluted in RBCs and medium to reach 1 mL of suspension at 5% hematocrit. Four aliquots of 200 μL were serially diluted and processed as described in the section “Validation under drug-free conditions.”

### *In vivo* comparison of LDA and regrowth assay.

Humanized mice were treated with different artesunate regiments as described above. Prior to each treatment and after 8, 24, 48, 72, 96 and 168 h, 40 μL of blood was collected using a heparinized capillary and transferred into 10 mL of 37°C preheated medium. The suspension was centrifuged (5 min at 290 × *g*) and the supernatant discarded.

For the *ex vivo* regrowth assay (ERA), 10-μL pellets of the washed mouse blood sample were processed as described above for the IRA.

For the *ex vivo* LDA, 10-μL pellets of the washed mouse blood sample were diluted in RBCs and medium to reach 500 μL of suspension at 5% hematocrit. Aliquots of 75 or 200 μL were serially diluted (3- to 4-fold), enabling four or two technical replicates, respectively. Otherwise, plates were processed as described for the *in vitro* LDA.

### Estimating parasite viability.

To estimate parasite viability, we fitted the data from the regrowth assay with a mathematical model of parasite growth as described in reference 19. Briefly, this model assumes that parasites from all samples belonging to a single mouse, *i*, grow in culture exponentially at rate $g_{i}$. Hence, the number of viable parasites in a sample collected from the mouse at time $T_{j}$, which has been in the culture for time *t*, is

(1) $$P_{\mathrm{ij}}\left(t\right)=\;B_{0i}\;\exp-n_{i}t\;+\;f_{\mathrm{ij}}V_{0i}\;\exp\;g_{i}t,$$

where $V_{0i}$ is the viable parasitemia in mouse *i* before treatment (i.e., the 0-h sample from each mouse), and $f_{\mathrm{ij}}$ is the fold change in viable parasitemia in each sample collected at time $T_{j}$ from mouse *i* after treatment. The value $B_{0i}\;$ is a background value from the measurement method (19), and $n_{i}$ is the rate at which the background (off-target) staining declines in the cultures. The human viability data were estimated in reference 19 using the method that we just described. For the mouse data, all the parameters in this model were estimated by fitting this model to the data on the regrowth data described above, and the fitted model provided an estimate of the change in viability in each sample from each mouse (Fig. S2).

### Measuring DHA concentrations.

A volume of 25 μL of whole-blood samples was collected via the tail vein from individual mice at 0.75, 1.5, 3, 8, 26, 50, and 74 h from the 50-mg/kg 4-daily-dose group and at 0.75, 1.5, 3, and 8 h from the 200-mg/kg single-dose group. The blood samples were immediately suspended in 25 μL of a stabilizing solution containing 21 mM potassium oxalate (223425; Sigma-Aldrich), 115 mM sodium fluoride (201154; Sigma-Aldrich), 18 mM deferoxamine mesylate (D9533; Sigma-Aldrich), and 80 mM potassium dichromate (207802; Sigma-Aldrich). Samples were vortexed for 20 s before being stored at −80°C. DHA concentrations were quantified within 2 weeks after collection by high-performance liquid chromatography-tandem mass spectrometry (HPLC-MS/MS).

### Pharmacokinetic model.

We used a one-compartment model with first order absorption to fit dihydroartemisinin (DHA) concentration measured in the human volunteer study (25, 26) and four mice from this study. Briefly, the model is defined as follows:

(2) C(t)=D×kaVd/F(ka−CLVd) ×[exp⁡(−CLVd×(t−tlag))− exp⁡(−ka×(t−tlag)) ],

where $V_{d}/F$ (L) is the apparent volume of distribution, $t_{\text{lag}}\;(\text{h})$ is the lag time between administration and absorption, CL/F (L/h) is the apparent drug clearance, $k_{a}$ is the rate of drug absorption, and *D* is the dose given to each subject. The model was fit with and without a lag for the absorption. The absorption was too fast to be observed in mice, and the one-compartment open model without a lag term fit the murine data best. Hence for mice, the factors $t_{\text{lag}}=0$ and $k_{a}$ are large enough such that equation 2 reduces to the following form:

C(t)=DVdF ×exp⁡(−CLVd×t) 

A one-compartment model with a first-order absorption and a lag, fit the human data best. The dose *D* of DHA was determined based on the mass of sodium artesunate administered to each individual/mouse multiplied by the scaling factor 0.7397 (ratio between DHA and artesunate molecular weights).

### Age-structured model of parasite growth.

To model the cyclical growth of P. falciparum parasites, we used a previously published age-structured model of parasite growth, which tracks the distribution of parasite stages developing in time (40):

(3) $$v_{T}\left(a,T\right)+v_{a}\left(a,T\right)=0\;$$

where, *v* is the density of parasites at time *T* postinfection and age *a*. In this equation, subscripts indicate the partial derivative of *v* with respect to the subscript variable. The boundary condition v(0,T)=PMF× v(A,T) indicates that mature parasites of age *A* produce PMF times more parasites of age 0 h, and the initial condition is given by $v\left(a,0\right)=P_{0}\times f(a;\sigma ,\mu )$, where f(a;σ,μ) is the probability density function of a truncated normal distribution over (0,A) with mean μ and standard deviation σ. The constant PMF is the parasite multiplication factor. Since parasites older than approximately 24 h of age are sequestered and no longer in circulation, the total number of circulating viable parasites (*V*) at any time is given by

$$V(T)=\;\overset{a_{s}}{\underset{0}{\int }}v\left(a,T\right)da$$

where $a_{s}$ is the age that parasites sequester. Importantly, as has been reported previously, the Plasmodium falciparum 3D7 strain used in these studies has an approximately 40-h blood-stage cycle (41, 42), so the maximum age of parasites is taken to be A=40h.

### Pharmacodynamic model.

We extended an existing age-structured model of parasite growth (43) to include drug killing of parasites. The age-structured model was used to capture the differential activity of artesunate on different life stages (44, 45). The model has two parasite compartments: one for viable parasites and one for nonviable parasites. The model of drug killing of viable parasites and host removal of nonviable parasites after treatment is defined as follows:

$$v_{t}\left(a,t\right)+v_{a}\left(a,t\right)=\;-\kappa \left(a,C(t)\right)\times v\left(a,t\right)$$

(4) $$u_{t}\;\left(a,t\right)=\kappa \left(a,C\left(t\right)\right)\times v\left(a,t\right)-\delta \times u\left(a,t\right)\;\text{for}\;a>0$$

where v(a,t) and u(a,t) are the density of viable and nonviable parasites of age *a* at time *t* posttreatment, respectively. The function κ is the killing rate of parasites, which depends on the DHA concentration, C(t), and the parasites’ age, *a*. The parameter δ is the rate of host removal of nonviable parasites. The total concentrations of circulating V(t) viable and nonviable U(t) parasites at time *t* are defined as follows:

$$V\left(t\right)=\;\overset{a_{S}}{\underset{0}{\int }}v\left(a,t\right)da$$

$$U\left(t\right)=\;\overset{a_{S}}{\underset{0}{\int }}u\left(a,t\right)da$$

where $a_{s}$ is the parasite sequestration age. We assume that the parasites are not sequestering in mice, in which case, $a_{s}=A$ (the length of the parasite blood-stage life cycle), and that parasites sequester at $a_{s}=A/2$ in humans (46). To account for the high susceptibility of very young ring stage parasites to dihydroartemisinin (44), we define the boundary condition such that a proportion of newly produced viable ring stages (of age zero) immediately become nonviable

v(0,t)=(1−E(t)) ×PMF×v(A,t)

and thus, the nonviable parasite density of age zero (i.e., a=0) has the same form as the above differential equation for u(a,t), but with an additional inflow of nonviable early ring stages, i.e.,

$$\frac{\partial u\left(0,t\right)}{\partial t}=E\left(t\right)\times \text{PMF}\times v\left(A,t\right)+\kappa \left(0,C\left(t\right)\right)\times v\left(0,t\right)-\delta \;\times u(0,t)$$

where

$$E\left(t\right)=\;\frac{C{\left(t\right)}^{\gamma }}{C{\left(t\right)}^{\gamma }+{\text{EC}}_{50}\gamma \;}$$

The maximum killing rate m(a) is a function of the parasite age since very-late-stage parasites are not killed by treatment (47). Hence, we define the killing function κ(a,t) as follows:

κ(a,t)=K(a)×E(t)

$$K\left(a\right)=\;\{\begin{array}{c} E_{\text{max}},\;a<a_{\text{schiz}}\\ 0,\;a\geq a_{\text{schiz}} \end{array}$$

As in previous studies, the age above which late-stage parasites survive treatment $\left(a_{\text{schiz}}\right)$ is taken to be the final 4 h of a 48-h life cycle (48, 49). The malaria parasite strain used in this study has a 40-h life cycle (42, 46), and we take $a_{\text{schiz}}$ as the last 4/48th of the life cycle.

### Model fitting and model comparison.

We used a least-square approach when fitting the *ex vivo* regrowth assay to estimate the concentration of viable parasites. We used a nonlinear mixed-effects approach when fitting the DHA concentration data with the pharmacokinetic model above in both mice and humans, with random effects for all parameters except for the apparent volume of distribution. DHA concentration measurements that were below detection limits were handled as described in section B in the supplemental material. When fitting the pharmacodynamic model, we first fit the pretreatment parasite concentration data for each human subject with the parasite growth model (equation 3) and estimated the distribution of parasite stages at the time of treatment and the parasite multiplication factor (PMF) when drug is not present. For the mice, pretreatment parasitemia curves were not detailed enough to allow fitting to estimate parasite life stage distributions and PM: instead, we estimated the PMF for each mouse from the *in vivo* recrudescence curves after treatment (fitting a tobit regression model using R package *censReg*) (Fig. 3), and we estimated the life stage distribution using the life stages from flow cytometric analysis (outlined in section C in the supplemental material). Once we obtained the fitted functional forms of the DHA concentration over time for each human subject and mouse (parameters in Table 1), the PMF with no drug present for each subject or mouse and the life stage distributions at the time of treatment for each subject or mouse (Fig. S5 and S6), we fitted the pharmacodynamic model in equation 4 to the viability data (from the regrowth assay) and the total circulating parasite concentration (humans)/parasitemia (mice) data using a maximum likelihood approach with censoring of data below the limit of detection (likelihood function and details of censoring in section B2 in the supplemental material). The list of parameters estimated from this approach is shown in Table 2. We fit this model to all data on parasite viability and total circulating parasitemia from mice and humans simultaneously with a variety of parameters constrained to be equivalent between mice and humans, in order to test which parameters, if any, were required to be different between the two hosts in order to best explain the data. We compared these models using the likelihood ratio test (for nested models) and the Akaike information criterion (AIC). A model with fewer parameters was deemed a better fit of the data unless the likelihood ratio test yielded a *P* value of >0.05 (for nested models) or the difference in AIC of >10 for nonnested models.

### Data availability.

All data and code will be made available upon reasonable request to the authors.
