# Supplementary material for: Parasite Viability as a Measure of In Vivo Drug Activity in Preclinical and Early Clinical Antimalarial Drug Assessment
Source: Antimicrob Agents Chemother. 2022 Jun 21;66(7):e00114-22. doi: 10.1128/aac.00114-22 (PMC9295577; doi:10.1128/aac.00114-22)
Supplement: Supplemental file 1 — Supplemental methods, Fig. S1 to S7, and Tables S1 to S3. Download aac.00114-22-s0001.pdf, PDF file, 1.7 MB [file aac.00114-22-s0001.pdf]

Supplementary Material

A. Limiting Dilution Assay vs Ex-vivo Regrowth Assay

We compared the parasites viability estimate from the regrowth assay (RA) to parasites viability estimated from a limiting dilution assay (LDA).

1. Estimating parasite viability from the limiting dilution assay data

We ran a LDA experiment for estimating the viable parasite concentration C_T in blood samples collected at time T. The assay is run by using F-fold dilution, which are replicated r times. Assuming the expected frequency of viable parasites in the undiluted sample is P_1, the wells at the second dilution level are expected to have a viable parasite frequency of $P_2 = \frac{P_1}{F}$ and so forth up to the wells at the highest dilution level d that is expected to contain a parasite frequency of $P_d = \frac{P_1}{F^d}$. For each well cultured, if viable parasites have grown above the detection limit of the assay, the well is scored as positive for parasites. If not it is scored as negative.

Following references (1-3), the probability that there are no viable parasites in a given well at a dilution level d is $q_d = \exp(-C_T \times P_d)$, where P_d is the number of parasites in the well. For the r_d replicates at dilution level d, if n_d is the number of wells with negative score, then the likelihood of the observing the results of the assay is

$$L = \prod_{d=1}^{D} \binom{r_d}{n_d} \times \left(1 - q_d^{r_d - n_d}\right) \times q_d^{n_d}.$$

Given that the parameters r_d, n_d and P_d are fixed by the design of the assay, we minimise the negative log of this likelihood function and estimate the concentration C_T of viable parasites in the undiluted sample, using the function lsqnonlin in MATLAB (version 2021b).

2. Control study with non-treated parasites

We first run the comparison between the two methods using serial dilutions of untreated samples. More precisely, we start with infected red blood cell samples with known parasitemia which were collected from an in vitro culture infected with *Pf3D7*$^{0087/N9}$. Since the cultures were not treated, all parasites counted are assumed to be alive. These initial samples are then serially diluted 10-fold, 6 times. For each of the 14 samples thus obtained, we take a first sample to perform a duplicate 4-fold LDA and a second sample to run the RA in duplicate. We found the two methods agreed strongly on untreated samples of parasite concentration and accurately estimate the known parasitemia (Figure 1).

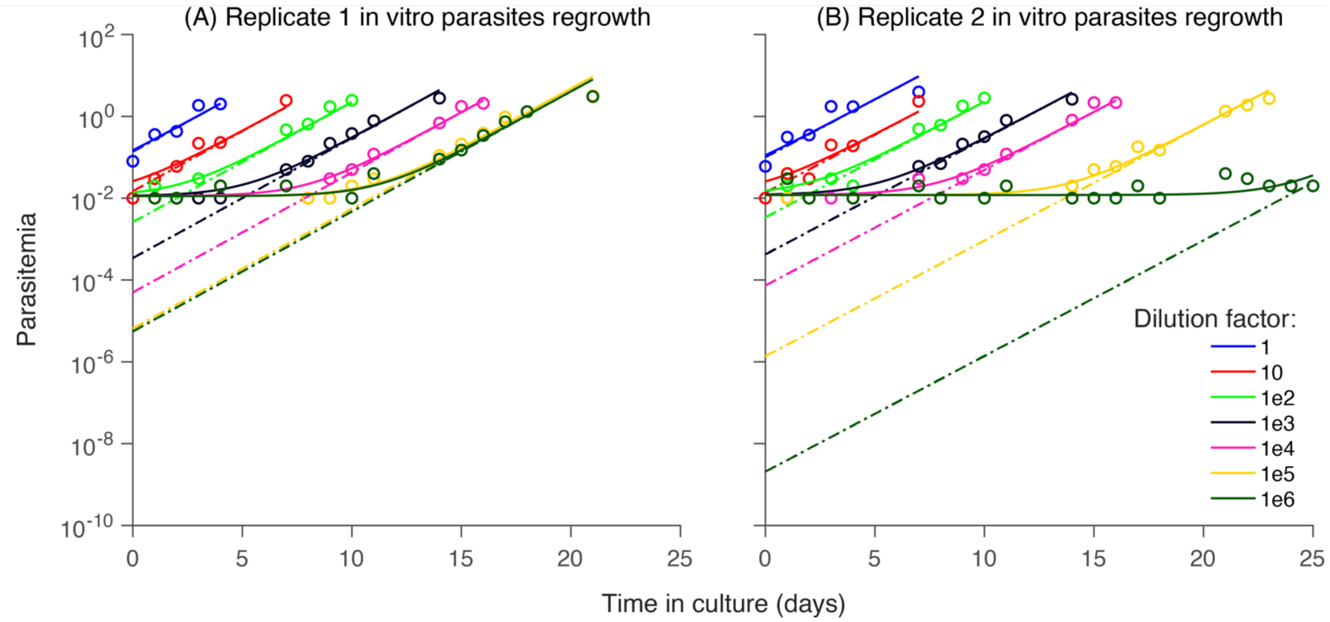

Figure S1 Fit of the parasite growth of untreated samples grown in vitro, as per the regrowth assay. Two replicate samples were run with six serial dilutions of each sample (the original concentration and the 6 times 10-fold dilution from 1 to 6). Each colour represents a dilution level. The circles are the measured parasitemia during the regrowth assay. The continuous lines are the models of the parasite regrowth, and the dashed lines are the projection of the growth models to estimate the initial concentration of viable parasites at the start of the culture.

3. Estimating parasite viability in treated mice

We estimated the concentration of viable parasites in samples collected from mice at 0, 24, 48, 72, 96 and 168 hours following artesunate treatment. These samples come from n = 14 mice. We use a duplicate 4-fold dilution for 10 mice and a threefold dilution repeated 4 times for 4 mice.

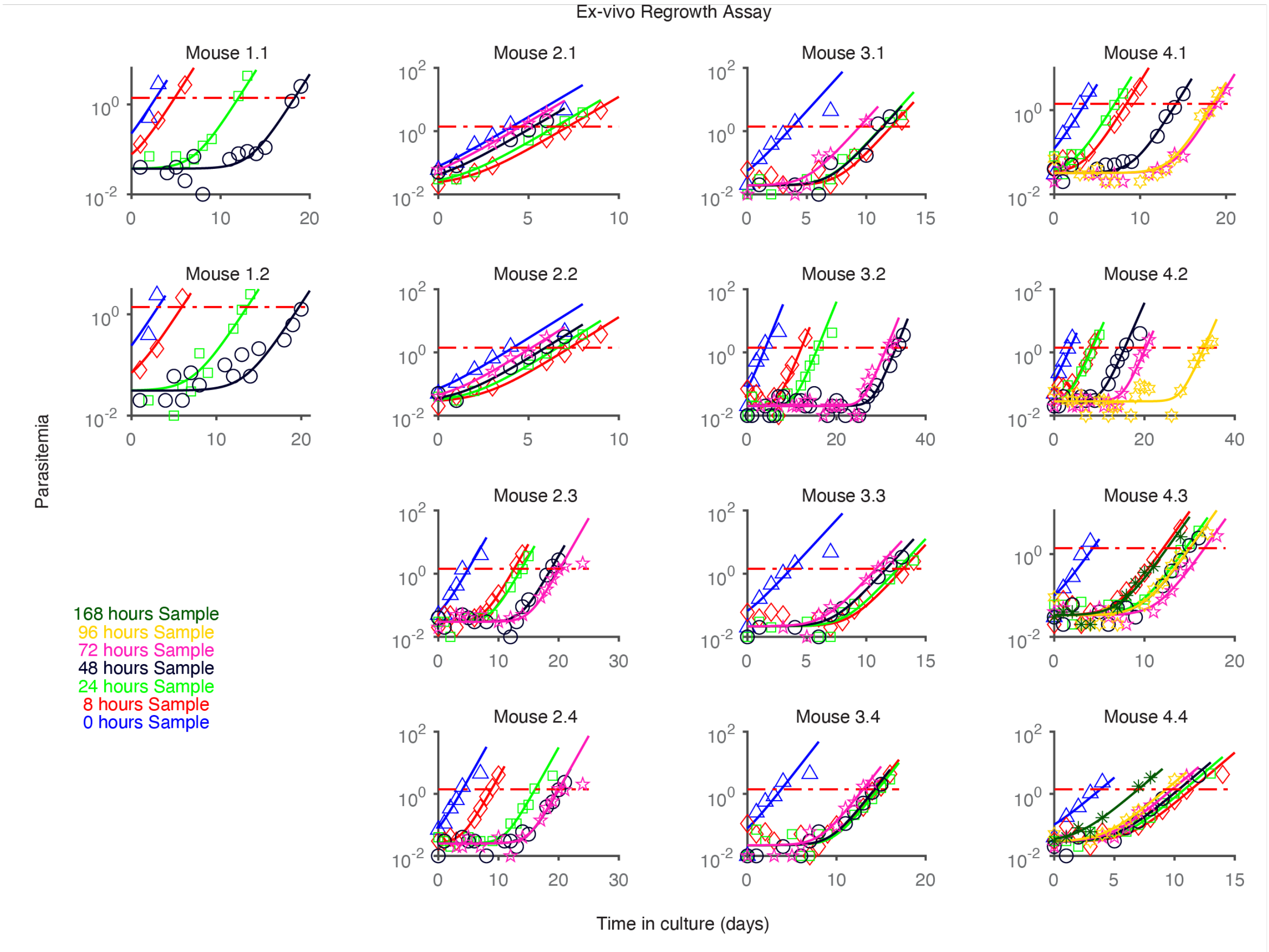

Figure S2. *Ex-vivo* parasite regrowth assay for sample from 14 mice. The continuous lines are the growth profiles of viable parasites collected at 0, 8, 24, 48, 72, 96 and 168 hours after treatment. Mouse i.j is the j-th mouse treated from experiment i.

B. PK Fitting

Limit of detection for PK model fitting

When fitting the pharmacokinetic model in equation 2 to the DHA concentration, if the data was below the LOD then the data was set to the limit of detection, and if the model estimate was also below the limit of detection the model also returned the LOD value. Thus, when the model and the data were below detection the model was assumed completely consistent with the data, but when the model or data was above detection while the other was below detection the model was penalised. LOD was 1ng/mL and 10 ng/mL in mice and human respectively.

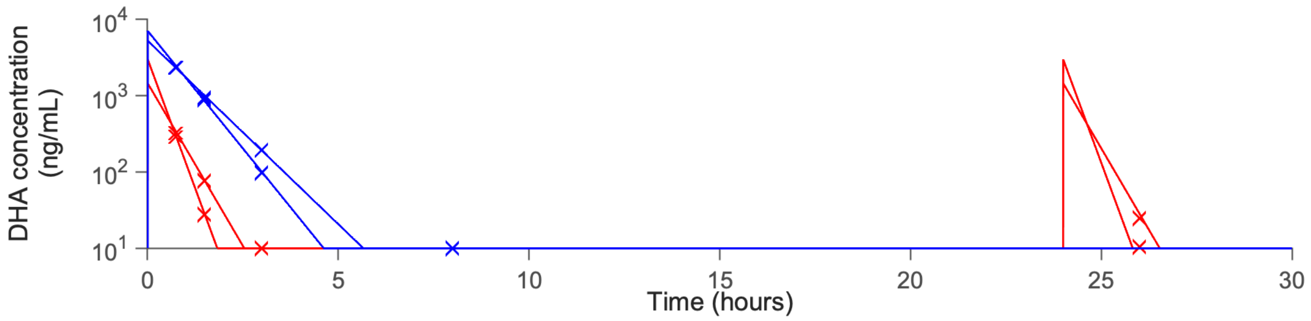

Figure S3. PK profiles fitted from drug concentration measured in n = 4 mice treated with single dose 200 mg (n = 2, in blue) and 4 days × 50 mg/day dose (in red). The crosses are the drug concentration measured in the mice plasma. The lines are the fitted PK models.

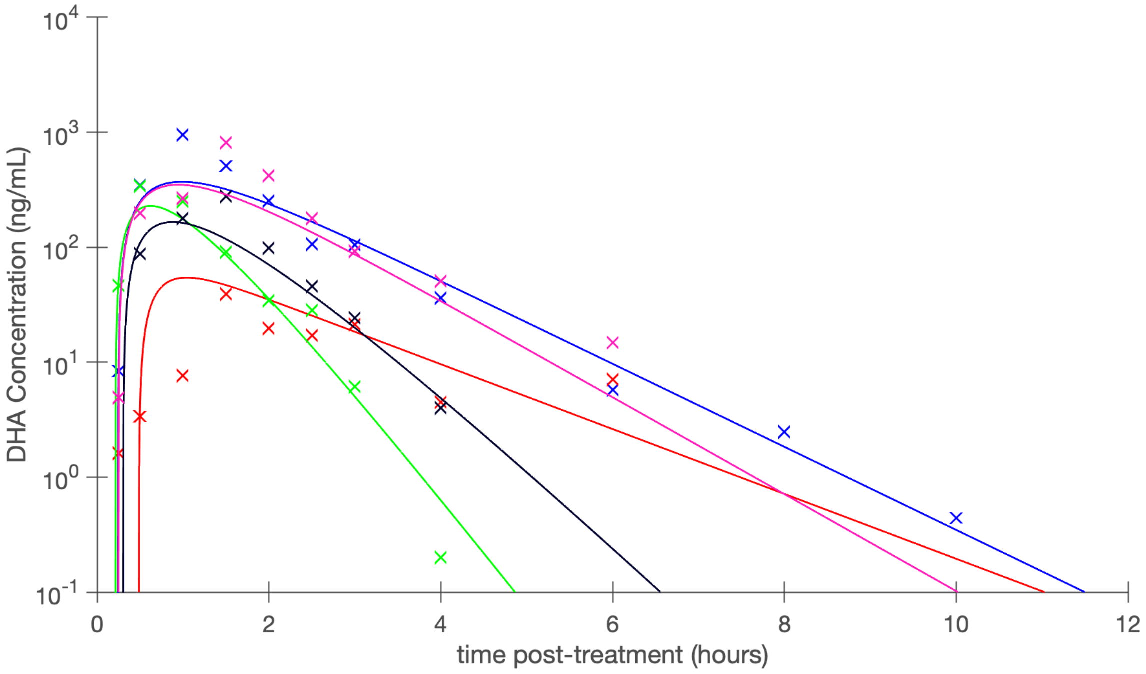

Figure S4. DHA pharmacokinetic profiles for five human volunteers infected with Plasmodium falciparum. Each colour represents one volunteer. The crosses are the measured data, and the black lines are the fitted PK model.

Parameter (units)	Non-Compartmental analysis* Population mean (CV)		One Compartment model**	
	ART-S	ART-R	Population Estimate (95% CI)	BSV (Standard deviation)
$CL/F\ (L\ h^{-1})$	157.9 (89.9)	133.2 (33.2)	254.5 (144.5, 364.5)	167
$V/F\ (L)$	310.3 (119.4)	221.1 (53.6)	165.9 (132.7, 199.2)	-
$k_a\ (h^{-1})$			2.29 (1.17, 3.42)	0.94
t_{lag}			0.62 (0.4, 0.83)	0.33
Dose-Normalised $AUC_{0-\infty}$ $(\mu g\ h\ L^{-1} mg^{-1})$	7.5 (33.2)	6.3 (89.9)	5.34 (3.83, 6.52) [a]	

Table S1 Comparison of pharmacokinetic parameters for DHA from the non-compartmental analysis in the original human volunteer infection study (4) and those presented here from a one compartmental model. The parameters: k_a absorption rate, F bioavailability, V/F apparent volume of distribution, t_{lag} lag-time, BSV Between Subject Variability, CV coefficient of variation. *Parameters estimates from (4) based on analysis of all subjects included in the original trial (n = 9 for ART-S and n = 13 for ART-R). ** Compartmental estimates based on a sub-population of the sample in (4) for whom viability was also assessed. Here the PK in both ART-R and ART-S infections were fitted together. [a] Average from the 10 volunteers. The confidence interval was computed using bootstrap.

C. Estimating the life-stage distribution at the time of treatment

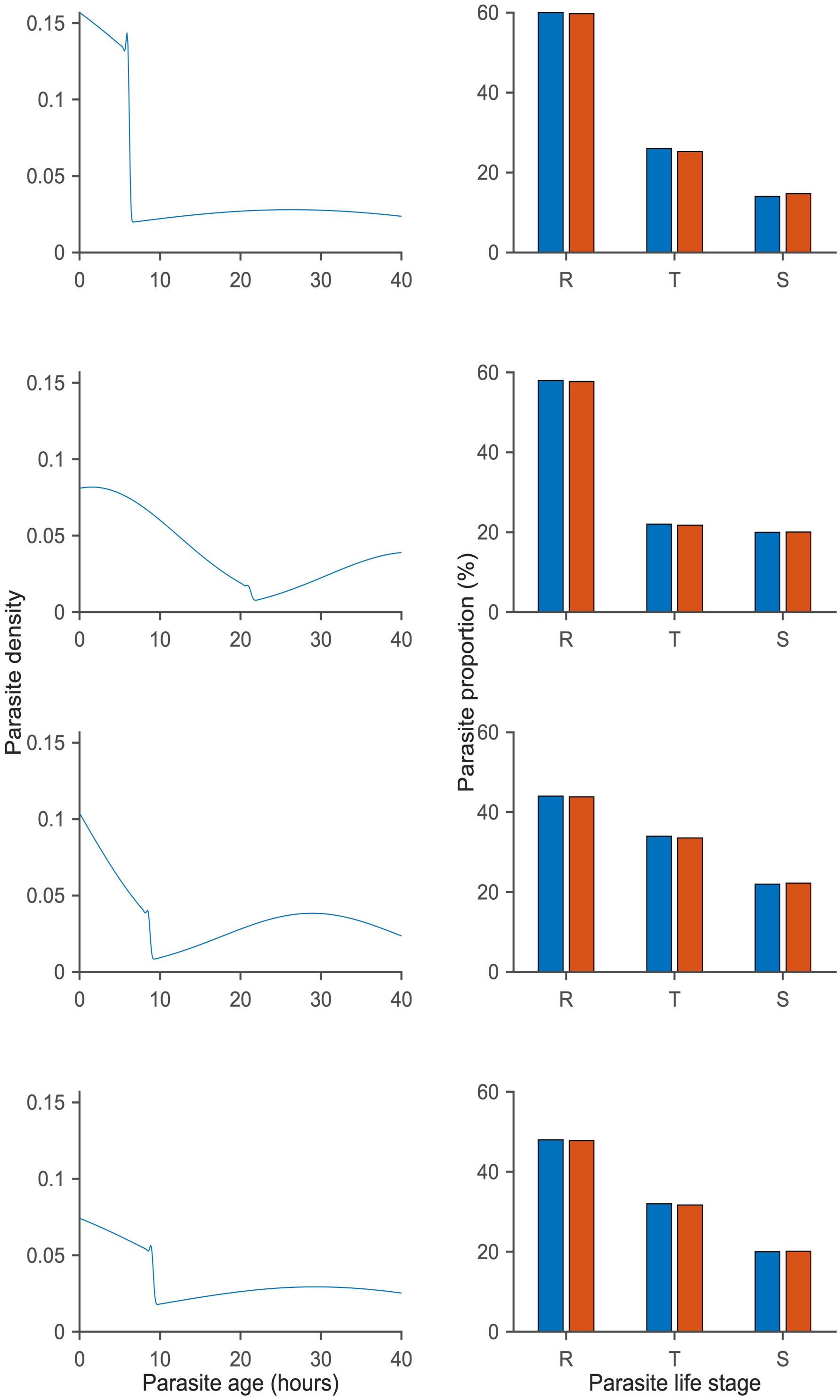

Figure S5. Parasite distribution at treatment time in each mouse (from experiment 4). The left panels are the estimated parasite distributions for each mouse. The right panels show the comparison of the model (blue) of the distribution of life-stages (R: Ring-stage, T: Trophozoite-stage, S: Schizont-stage) in each mouse (left panel) with the observed (red) life-cycle distribution determined by microscopy.

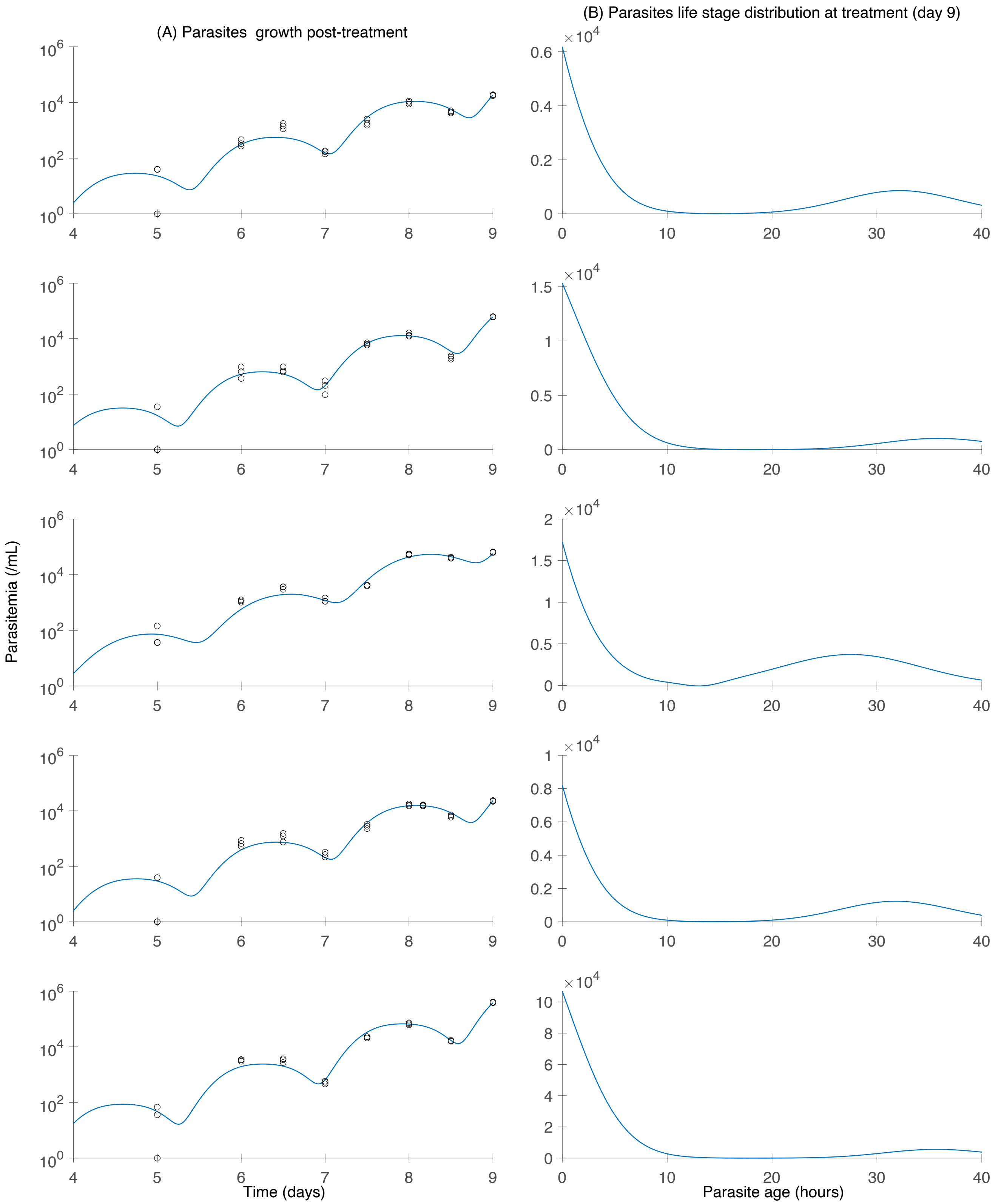

Figure S6. Parasite growth profiles post-treatment and parasite distributions at treatment time in five human volunteers. Each row represents data from a volunteer. In the left panels, the points are the measured parasitemia and the lines are models of parasites growth. The right panel shows life stage distribution estimated from the parasite growth model at treatment time (day 9).

D. Fitting the Pharmacodynamic model

We use a maximum likelihood approach with a likelihood function that has two parts. A censored part, which is a log-normal cumulative distribution function and an uncensored part, which is a log-normal probability density function.

For the human, the censoring upper bound is 32 parasites/mL and 20 parasites/mL for the circulating and viable parasites, respectively. The upper bound censoring is 0.01% parasitemia for circulating parasites in mice and we do not have censoring for viable parasites in mice.

The most complex model has a total of 14 parameters with 8 pharmacodynamic parameters (two sets of (E_{max}, EC_{50}, γ, δ) for mice and humans) and 6 standard deviation parameters related to the probability distribution in the likelihood function. We always allow δ to differ between mice and humans and we used a likelihood ratio test to compare nested models to determine which of the PD parameters (E_{max}, EC_{50}, γ) were different between mice and humans. We assume first that these three PD parameters are the same between mice and humans and we test if increasing model complexity significantly improves the fit (fig. S7). We find two best-fitting models, which were not nested had less than 10 AIC points different (tab. S3), a model where E_{max} is different between mice and humans (but not EC_{50}) and a model where EC_{50} is different between mice and humans.

Model	#Parameters	df	Log likelihood	ΔG^2	Critical value
Reduced: E_{max}, EC_{50}, γ	11	1	-397.24	74.77	3.84
Extended: E_{max}, EC_{50H}, EC_{50M}, γ	12		-359.85		
We reject the reduced model					
Reduced: E_{max}, EC_{50}, γ	11	1	-397.24	76.52	3.84
Extended: E_{maxH}, E_{maxM}, EC_{50}, γ	12		-358.98		
We reject the reduced model					
Reduced: E_{max}, EC_{50}, γ	11	1	-397.24	3.7	3.84
Extended: E_{max}, EC_{50}, γ_H, γ_M	12		-395.39		
We reject the extended model					
Reduced: E_{max}, EC_{50H}, EC_{50M}, γ	12	1	-359.85	1.8	3.84
Extended: E_{maxH}, E_{maxM}, EC_{50H}, EC_{50M}, γ	13		-358.95		
We reject the extended model					
Reduced: E_{maxH}, E_{maxM}, EC_{50}, γ	12	1	-358.98	0.06	3.84
Extended: E_{maxH}, E_{maxM}, EC_{50H}, EC_{50M}, γ	13		-358.95		
We reject the extended model					
Reduced: E_{maxH}, E_{maxM}, EC_{50}, γ	12	1	-358.98	0.82	3.84
Extended: E_{maxH}, E_{maxM}, EC_{50}, γ_H, γ_M	13		-358.57		
We reject the extended model					
Reduced: E_{max}, EC_{50H}, EC_{50M}, γ	12	1	-359.85	2.74	3.84
Extended: E_{max}, EC_{50H}, EC_{50M}, γ_H, γ_M	13		-358.48		
We reject the extended model					
Reduced: E_{maxH}, E_{maxM}, EC_{50}, γ	12	2	-359.85	3.72	5.99
Extended: E_{maxH}, E_{maxM}, EC_{50H}, EC_{50M}, γ_H, γ_M	14		-357.99		
We reject the extended model					
Reduced: E_{max}, EC_{50H}, EC_{50M}, γ	12	2	-358.98	1.98	5.99
Extended: E_{maxH}, E_{maxM}, EC_{50H}, EC_{50M}, γ_H, γ_M	14		-357.99		
We reject the extended model					

Table S2 Model Selection with loglikelihood ratio test. E_{max} maximum killing rate; EC_{50} drug concentration to reach half maximum killing rate; γ hill coefficient. The parameters with underscore M and H are parameters specific to mice and humans, respectively.

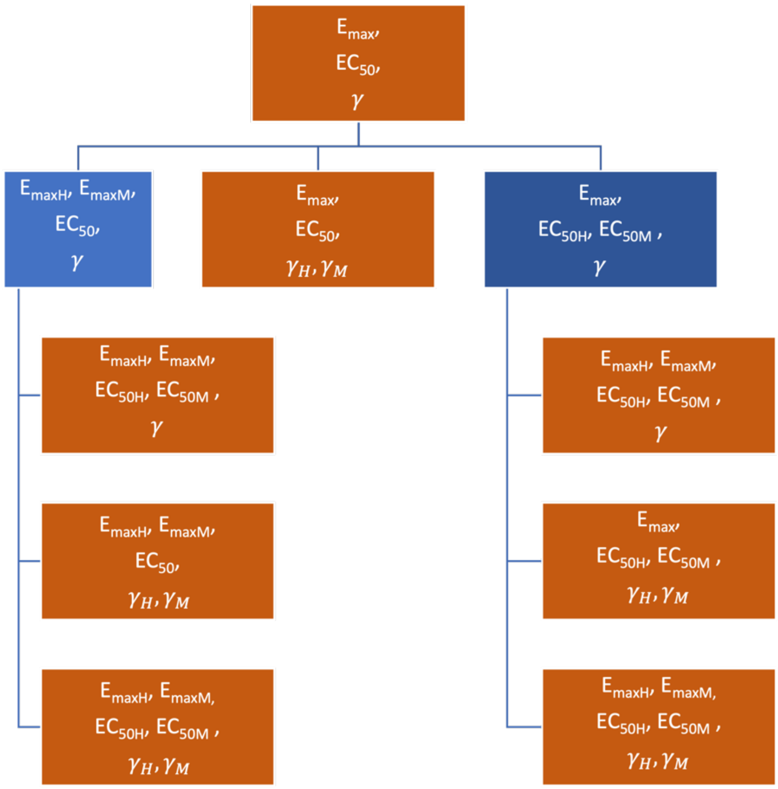

Figure S7 Model hierarchy. Each set of parameters represent a model. The models in orange are rejected models. E_{max} maximum killing rate; EC_{50} drug concentration to reach half maximum killing rate; γ hill coefficient. The parameters with underscore M and H are parameters specific to mice and humans, respectively.

Models	#Parameters	Loglikelihood	AIC	ΔAIC
E_{maxH}, E_{maxM}, EC_{50H}, EC_{50M}, γ_H, γ_M	14	-357.99	743.98	2.02
E_{maxH}, E_{maxM}, EC_{50}, γ_H, γ_M	13	-358.57	743.15	1.19
E_{max}, EC_{50H}, EC_{50M}, γ_H, γ_M	13	-358.48	742.97	1.01
E_{maxH}, E_{maxM}, EC_{50H}, EC_{50M}, γ	13	-358.95	743.91	1.95
E_{maxH}, E_{maxM}, EC_{50}, γ	12	-358.98	741.96	0.00
E_{max}, EC_{50}, γ_H, γ_M	12	-395.39	814.79	72.83
E_{max}, EC_{50H}, EC_{50M}, γ	12	-359.85	743.70	1.74
E_{max}, EC_{50}, γ	11	-397.24	816.48	74.52

Table S3 Model Selection with AIC. E_{max} maximum killing rate; EC_{50} drug concentration to reach half maximum killing rate; γ hill coefficient. The parameters with underscore M and H are parameters specific to mice and humans, respectively. The AIC difference between the two best models is less than 10. AIC = -2× log-likelihood + 2× #Parameters.

References

1. L. E. Myers, L. J. McQuay, F. B. Hollinger, Dilution assay statistics. *J Clin Microbiol* **32**, 732-739 (1994).
2. T. Bonnefoix, P. Bonnefoix, P. Verdiel, J. J. Sotto, Fitting limiting dilution experiments with generalized linear models results in a test of the single-hit Poisson assumption. *J Immunol Methods* **194**, 113-119 (1996).
3. Y. F. Hu, G. K. Smyth, ELDA: Extreme limiting dilution analysis for comparing depleted and enriched populations in stem cell and other assays. *J Immunol Methods* **347**, 70-78 (2009).
4. R. E. Watts *et al.*, Safety and parasite clearance of artemisinin-resistant Plasmodium falciparum infection: A pilot and a randomised volunteer infection study in Australia. *PLoS Med* **17**, e1003203 (2020).
